# Dialysis peritonitis in a patient with chronic kidney disease and multiple comorbidities

**DOI:** 10.1186/1471-2334-13-S1-P34

**Published:** 2013-12-16

**Authors:** Oana Streinu-Cercel, Anca Streinu-Cercel, Ioana Berciu, Alina Cristina Neguț, Mihai Săndulescu, Maria Magdalena Moțoi, Adrian Streinu-Cercel

**Affiliations:** 1 Carol Davila University of Medicine and Pharmacy, Bucharest, Romania; 2 National Institute for Infectious Diseases “Prof. Dr. Matei Balş”, Bucharest, Romania

## Background

Patients with peritoneal catheters are at risk for developing infections with germs with altered antibiotic sensitivity, being classified as Carmeli 3 due to repeated invasive contact with the hospital system. A cloudy peritoneal fluid is oftentimes a sentinel sign of infection and medical and surgical management are generally required to clear infection and prevent subsequent reoccurrences.

## Case report

We report the case of a 70 year-old male patient, with chronic kidney disease under peritoneal dialysis, type II diabetes mellitus, cardiac insufficiency, arterial hypertension, dyslipidemia, bilateral carotid atheromatosis, grade 1 obesity, COPD, and history of repeated peritonitis, including a previous episode of sepsis with MSOF.

The presenting complaint, dating back two weeks, consisted of lower abdominal pain followed by two unformed stools; 12 hours later the dialysis fluid turned cloudy; 24 hours later the patient was admitted to a Nephrology Clinic. In the tenth day of symptomatology, the patient became drowsy and was transferred to our clinic, for infectious diseases management.

Clinical exam at admission revealed: mediocre clinical state, bilateral leg and dorsal hand edema, arterial tension 140/90 mmHg, pulse 80 bpm, abdomen distended through peritoneal dialysis, hepatomegaly.

Lab results showed inflammatory syndrome, slight anemia, nitrogen retention syndrome.

Peritoneal fluid cultures grew *Rothia mucilaginosa* and the patient mentioned recent dentist treatment with full-mouth prosthetic rehabilitation. We performed a complete dental exam and cultures from: gingival sulcus fluid, dental plaque, tongue; results came out positive for *Enterococcus faecalis* and *Candida* spp and peritoneal fluid cultures grew *Candida lipolytica*. Over the course of hospital admission, the peritoneal count rose to over 4000 cells/cmm, and the patient presented fever, chills, obnubilation.

Under treatment with meropenem, linezolid, voriconazole and peritoneal instillations with vancomycin, the patient’s evolution was favorable.

Due to the isolation of *Candida lipolytica*, together with the nephrologist and the surgeon, on the 23^rd^ day of evolution a subclavian hemodialysis catheter was placed and on the 25^th^ day, the peritoneal catheter was removed (cultures positive for *Candida lipolytica*). For the long term management of the kidney disease, a hemodialysis fistula was performed.

## Conclusion

The clinical evolution corroborated the initial suspicion of dialysis peritonitis of mixed etiology, fungal and *Rothia* spp. Close interdisciplinary collaboration between the infectious disease specialist and the nephrologist is mandatory in order to conduct a proper treatment.

